# Nurse managers’ attributes to promote change in their wards: a qualitative study

**DOI:** 10.1002/nop2.87

**Published:** 2017-07-07

**Authors:** Yoshimi Kodama, Hiroki Fukahori

**Affiliations:** ^1^ Department of System Management in Nursing Graduate School of Health Care Sciences Tokyo Medical and Dental University 1‐5‐45, Yushima Bunkyo‐ku Tokyo 113‐8519 Japan

**Keywords:** change process, leadership, nurse managers, qualitative research

## Abstract

**Aim:**

The aim of this study was to explore the processes that nurse managers use to promote change in their wards.

**Design:**

Qualitative research.

**Methods:**

A grounded theory approach was used. Participants were 23 nurse managers and 17 nurses in Japan. Interviews were conducted between March 2014 – December 2015. Mainly, nurse managers’ data was analysed.

**Results:**

The change process led by the nurse managers was depicted as a four‐phased process of “having beliefs and empathizing with staff nurses to achieve goals explored by self.” Four attributes of nurse managers, “having both micro and macro perspectives,” “respecting own beliefs and external standards,” “being proactive,” and “having empathy for staff nurses,” were identified as indispensable factors promoting change in their wards.

Nursing administrators should support the cultivation of nurse managers’ attributes for successful change.

## 1 INTRODUCTION

Nurse managers as first‐line leaders have a responsibility to induce changes in the clinical environment. Recently, this responsibility of nurse managers has become more important than ever due to demands for rationalization, cost cuttings, advancements in medical technology, and reduced lengths of hospital stay (Institute of Medicine, 2011; Japanese Nursing Association, 2016). Nurse managers need to exercise leadership to undertake this responsibility.

For this responsibility, various strategies have been implemented to develop nurse managers’ abilities to promote change. Nurse manager leadership programs have been developed in some countries (Japanese Nursing Association, 2016; Wallis & Kennedy, 2013). A program in the US (Wallis & Kennedy, 2013), that included building transformational leadership, demonstrated positive effects on nursing quality and the work environment (Cummings et al., 2010). In Japan, strategies for developing leadership were developed, such as introducing certified nurse administrators (Japanese Nursing Association, 2016). Furthermore, periodic ward rotation of nurse managers is conventionally scheduled to improve nurse services, stimulate quality ward activity and encouraging nurses’ career development (Fujino & Nojima, 2005).

Through such efforts, previous research showed that nurse managers’ abilities to promote change should be further developed because they experience difficulty in conceptualizing unidentified problems (Yoshida et al., 2010), which is necessary before strategies for change are implemented.

### 1.1 Background

Cross‐disciplinary research, such as organizational studies, can provide important insights that nurse managers can consider to cultivate their leadership skills (Shanley, 2007). However, representative theories developed in organizational behaviour science, including Havelock's (1973) theory of change and Rogers’ (2003) innovation theory, are difficult to adapt to nurse managers’ daily practice. First, present change theories require nurse managers to apply highly abstract concepts in their daily practice. Yoshida et al. (2010, 2014) indicated that nurse managers experienced difficulty in adapting general or grand theories to clinical nursing situations. Second, representative theories mainly focus on top managers or project leader activity, rather than focusing on middle managers as change agents. Middle managers’ strategies for change are different from those of top managers or innovation project leaders because middle managers must make binding decisions in units with limited power or authority (Drucker, 1986). Therefore, in addition to existing change theories, tacit knowledge about how middle nurse managers promote change should be developed because we lack in‐depth understanding of how nurse managers as middle managers change clinical situations. The description of the change process led by nurse managers will contribute to understanding of experienced nursing managers’ skills, and this description should be disseminated.

Though there are several previous studies regarding nurse managers’ activities, these findings are difficult to use for nurse managers to lead change processes because these studies did not describe how nurse managers identified problems in their wards. Some previous literature on nursing leadership described nurse managers’ experiences in implementing change, such as improving nursing quality, and leading evidence‐based practice projects induced by organizational projects or decisions by upper management such as nurse executives (Bondas, 2009; Hayman, Wilkes, & Cioffi, 2008; Suhonen & Paasivaara, 2011). In these situations, problems to be solved are determined in advance. These studies revealed that nurse managers should empower staff nurses to ensure effective project management (Hayman, Wilkes, & Cioffi, 2008), and they played a role in creating a culture that improved nursing quality (Bondas, 2009; Salmela, Eriksson, & Fagerstrom, 2012). While these studies are helpful for nurse managers to share given project goals with staff nurses, these studies are not sufficient for deep understanding of the processes allowing nurse managers to recognize and analyse problems in their own wards. As above, nurse managers face challenges in analysing clinical situations and conceptualizing problems (Yoshida et al., 2010). Therefore, it is important to develop knowledge about the processes nurse managers use to autonomously identify problems in their wards and promote changes.

## 2 THE STUDY

### 2.1 Aim

The study aimed to explore and describe the processes that Japanese nurse managers use to promote change in their wards.

### 2.2 Design

A grounded theory approach based on Interactionism (Corbin & Strauss, 2015) was chosen to explore and develop a theory concerning the processes of interest.

### 2.3 Study participants

Nurse managers and staff nurses (including assistant nurse managers) were recruited to understand interactions between them concerning change processes. A nurse manager has 24‐hour accountability and continuing employee development and directs, plans and coordinates the operational activities for the ward (Oku et al., 2010). An assistant nurse manager works under the nurse manager to help a nurse manager and has partial responsibility for management in the ward (Teraoka, 2011).

Theoretical sampling was used in this study. Data were collected to explore issues that emerged through data analysis, such as length of time as a manager, hospital characteristics, and relationships between nurse managers and assistant nurse managers.

The inclusion criteria for nurse managers were having more than 6 months experience as a manager and having implemented change in their wards. Criteria for staff nurses were having 1 year or more nursing experience and experience of the change process with nurse managers. The nursing directors of selected hospitals were asked to introduce the researchers to nurse managers who met study criteria. Potential participants were contacted directly. After being interviewed, nurse managers were asked to introduce the researchers to staff nurses familiar with the change process.

### 2.4 Data collection

One‐on‐one semi‐structured interviews were conducted between March 2014 – December 2015. Nurse managers were asked to recall a case wherein they initiated change in their wards; then staff nurses were asked how they felt about implementing change. An interview guide was developed and used (Table 1).

**Table 1:** Interview guide for a nurse manager

Could you tell me about your experiences when leading change in your ward? What problem did you change? When did you change the problem? Why did you feel the need to change the problem? How did you involve the staff nurses in the change process? Were there obstacles to the change process?

The participants were 23 nurse managers and 17 staff nurses (including assistant nurse managers).

### 2.5 Analysis

Mainly, nurse manager’s data were analysed. Staff nurses’ data were used in subsidiary analyses to understand interactions and ward situations more deeply. Data were analysed in conjunction with data collection utilizing the constant comparative method (Corbin & Strauss, 2015). Interview transcripts were read, reread, and analysed as data. The first author mainly analysed data. We divided the data into pieces by meaning chunks or line‐by‐line and assigned codes representing these meanings (Open coding). Data pieces were thought of as nurse managers, their actions and intentions, and the responses of staff nurses. We classified the codes into several categories according to their similarities.

The first author examined the interrelationship of categories while receiving advice from the second author about new ideas and coding. If necessary, we refined the name or sorting of categories (Axial coding). The first and second authors achieved maximum agreement about them.

The first author identified a core category, described a single storyline, and drew a figure showing the overall process. We refined them while receiving advice from the second author and we achieved maximum agreement about them.

### 2.6 Rigor

The rigour of this study was ensured using rich information through the recruitment of participants. In addition, certain strategies were conducted to enhance rigour as follows (Corbin & Strauss, 2015): (i) Memoing; (ii) Diagramming; (iii) Member checks; (iv) Peer debriefing with the second author and other researchers; (v) Documentation of the audit trail.

### 2.7 Ethics

This study was approved by the Institutional Review Board of Tokyo Medical and Dental University and Hospital (1720). Participants were assured of the voluntary nature of their participation and provided informed consent.

## 3 RESULTS

### 3.1 Participants’ characteristics and cases of promoting change

Nurse managers’ demographic data are reported in Table 2. Staff nurses consisted of eight RNs and nine assistant nurse managers, and their ages ranged from 20s–40s. The average nursing experience was 13.4 years.

**Table 2:** Participants’ demographic data (Only nurse managers)

ID	Age	Gender	Time as a manager (years)	Education level	Training for certified nurse administrators^a^	Area of practice (at the time of the case)	Hospital characteristics^b^	The number of beds
1	40s	F	7	Diploma	Yes	Medical	University hospital	About 800 beds
2	40s	F	10	Associate degree	Yes	Medical	University hospital	About 800 beds
3	50s	F	8	Diploma	No	Medical	University hospital	About 800 beds
4	30s	F	2.8	Diploma	Yes	Long term care/Rehabilitation	Sub‐acute rehabilitation hospital	About 600 beds
5	40s	F	3.2	Diploma	Yes	Long term care/Rehabilitation	Sub‐acute rehabilitation hospital	About 600 beds
6	30s	F	1.2	Diploma	No	Long term care/Rehabilitation	Sub‐acute rehabilitation hospital	About 600 beds
7	60s	F	25	Associate degree	No	Medical	Acute care hospital	About 300 beds
8	40s	F	3.3	Diploma	No	Medical/Surgical	Acute care hospital	About 300 beds
9	50s	F	6.3	Diploma	Yes	Medical/Surgical	Acute care hospital	About 300 beds
10	40s	F	10	Diploma	No	Surgical	Acute care hospital	About 300 beds
11	40s	F	2.6	Diploma	Yes	Surgical	Acute care hospital	About 300 beds
12	40s	F	7.3	Diploma	Yes	ICU	University hospital	About 800 beds
13	40s	F	3.7	Diploma	Yes	Medical/Surgical	Acute care hospital	Under 100 beds
14	40s	F	4.7	Diploma	Yes	Medical	Acute care hospital	About 300 beds
15	40s	F	9.7	Diploma	Yes	Medical	Acute care hospital	About 300 beds
16	40s	F	3.8	Associate degree	Yes	Medical	Acute care hospital	About 150 beds
17	40s	M	13	Diploma	Yes	Medical/Surgical/SCU	Acute care hospital	About 150 beds
18	30s	F	0.5	Diploma	Yes	Medical	Acute care hospital	About 150 beds
19	30s	F	0.5	Diploma	Yes	Medical/Surgical/SCU	Acute care hospital	About 150 beds
20	40s	F	3.8	Diploma	Yes	Palliative care	Acute care hospital	About 300 beds
21	30s	F	0.8	Diploma	No	Medical/Surgical	Acute care hospital	Under 100 beds
22	60s	F	10	Diploma	No	Medical/Rehabilitation	Acute care hospital	About 300 beds
23	50s	F	25	Diploma	No	Medical/Surgical	University hospital	About 800 beds

a The certified nurse administrator's system is designed to provide high quality organizational nursing services (Japanese Nursing Association, 2016) and this system includes a leadership and management program (Ibe et al., 2012).

b A university hospital is affiliated to a university providing advanced medicine. An acute care hospital provides advanced medical care including emergency treatment and surgery. In most cases, university hospitals and acute care hospitals hire more nurses per each patient to provide intensive care. A sub‐acute rehabilitation hospital focuses on rehabilitation and monitoring disease.

Topics of the cases that nurse managers discussed included low quality of nursing care (e.g. substandard end‐of‐life care, high rates of rehospitalization for heart failure and non‐compliance with protocols), poor work environment (e.g. high turnover rate, overtime work, bullying and inefficient patient care).

### 3.2 Overview of the results

**Figure 1:**
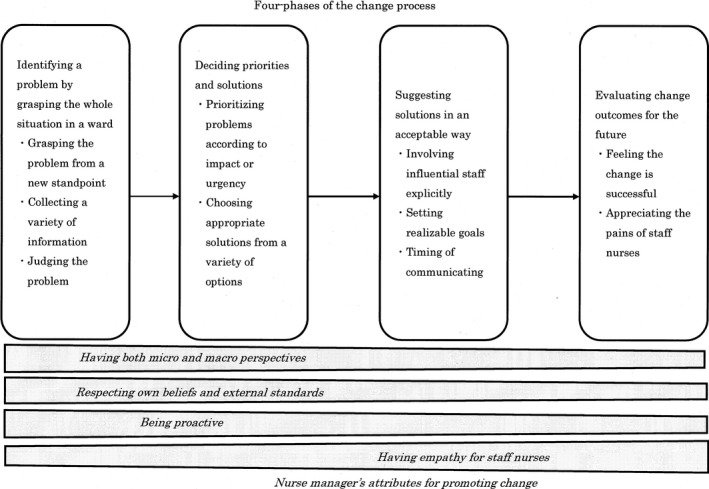
Having beliefs and empathizing with staff nurses to acheive goals explored by self

The change process led by the nurse managers was described as a four‐phased process of “having beliefs and empathizing with staff nurses to achieve goals explored by self” (Figure 1). Nurse managers struggled to change problems while maintaining their own beliefs and empathy with staff nurses. Through this whole process, nurse managers’ four attributes were identified to be indispensable to promote change on the wards. We first explain the four attributes before describing the change process in detail to help readers understand the effect of the attributes on the overall change process.

### 3.3 Nurse manager's attributes for promoting change

Nurse managers could promote changes in their wards using four attributes. The importance of each attribute varied for each phase. For example, in the first phase, three of the four attributes were especially important to promote change (Figure 1). The four attributes included “having both micro and macro perspectives,” “respecting own beliefs and external standards,” “being proactive” and “having empathy for staff nurses.”

#### 3.3.1 Having both micro and macro perspectives

Nurse managers made efforts to collect detailed information about wards such as the number of incidents, turnover rate, staff nurses’ characteristics, and relationships between nurses and doctors. Nurse managers analysed this information, and then they interpreted the context of events and inferred potential underlying causes. Nurse managers grasped the situation concerning the whole of the ward not just parts of it through the above assessment. These perspectives were useful for nurse managers to identify problems, develop strategies and evaluate outcomes for change. For example, a female nurse manager questioned a clinical situation involving many sudden changes in a patient's condition and interviewed some nurses about this situation. She deeply assessed the content of interviews and inferred that staff nurses’ physical assessment skills were quite low:

“After I was assigned this ward, a patient's condition suddenly deteriorated. Similar deteriorations occurred three times… I understood the situation that staff nurses did not have knowledge about emergency nursing, reported patients’ conditions to doctors too late and could not appropriately assess their vital signs, by interviewing staff nurses… eventually, I recognized that they did not have enough education (about acute care nursing)” (ID 7).

#### 3.3.2 Respecting own beliefs and external standards

When nurse managers judged what problems were in their wards or they evaluated outcomes of change, they performed multiple judgements, based on both their belief regarding nursing or leadership and standards such as the hospital's mission or the nursing association policy. Nurse managers’ beliefs were generated from their experiences as nurses or managers. For example, a male nurse manager had a strong belief about building a healthy work environment and his belief was consistent with his hospital's mission:

“My policy as manager is no working overtime… I kept my ward mission and hospital mission in mind. And then, I talked about a problem (overtime) for staff nurses” (ID17).

#### 3.3.3 Being proactive

The nurse managers predicted that the clinical situation would become worse, if they did not change the problems. This attribute enabled nurse managers to prioritize change and to have a longitudinal vision of their ward apart from the problem confronted at that time. In addition, nurse managers developed strategies to forestall staff nurses’ resistance to change.

A female nurse manager recognized that nursing shortages affected patient safety, that staff nurses suffered from the stress of this situation, and that in the future, this situation would lead to more turnover:

“I performed interviews with staff nurses until May. I understood the shortage of staff nurses in the ward (ICU)… If this situation continued, I thought that all staff nurses might leave the hospital” (ID12).

#### 3.3.4 Having empathy for staff nurses

Nurse managers made an effort to understand staff nurses’ feelings and respected their experience and pride in nursing care through the whole change process. Nurse managers recognized that empathy for staff nurses was needed for successful change. Nurse managers thought that they had a different standpoint from staff nurses because they had different responsibilities. Nurse managers’ main role was managing the ward; on the other hand, staff nurses took the role of providing patient care. Nurse managers understood that this difference led to disagreement between themselves and staff nurses about change. A female nurse manager said that when she desired to stop unnecessary routine weight measurement, she carefully communicated so that she would not hurt staff nurses’ pride or deny their efforts in routine work:

“(I was assigned new ward) staff nurses who provided unnecessary care(weight patients), every Saturday as routine work. This care was not provided as routine work in our hospital. If a nurse manager said to me that your care is a traditional way, I would be angry… I considered the manner of making this suggestion” (ID1).

Next, the four‐phased change process is described, emphasizing influences of the four nurse managers’ attributes.

### 3.4 Identifying a problem by grasping the whole situation in a ward

Almost all nurse managers had opportunities to find problems in their wards due to assignment to a new ward or hospital, and promotion. Nurse managers made an effort to collect information about wards through communication with staff nurses. Nurse managers collected detailed information from micro perspectives, assessed this information and deeply understood the ward from a macro perspective; thus, they could grasp the ward situation. Nurse managers compared the actual ward situation with the ideal one inferred from their beliefs or the hospital mission and identified the problem.

#### 3.4.1 Grasping the problem from a new standpoint

When almost all nurse managers were assigned to a new ward, transitioned to a new hospital, or were promoted from assistant nurse managers to manager, they questioned assumptions about conventional ways; that is, these nurse managers perceived the situation from a new standpoint. When nurse managers were assigned from other wards or hospitals to a new ward, differences between the new ward and the previous ward were highlighted. In this research, a nurse manager said that a nurse manager who belonged to the same ward for a long time followed a traditional way, so nurse managers’ assignments to new wards should be useful to change the wards:

“Ward rotation provides new scenery. I wondered why staff nurses acted this way… (a nurse manager) should do ward rotation, and avoid developing entrenched habits.” (ID1)

#### 3.4.2 Collecting a variety of information

Almost all nurse managers struggled to collect a variety of information from different sources to understand the ward situation in detail. Information sources included many medical staff, doctors in wards, or risk managers in hospitals, and data such as turnover rate. Especially, nurse managers gathered much information through meeting with assistant nurse managers. To understand staff nurses’ true feelings about the ward situation, nurse managers made efforts to build relationships with staff nurses. An exception was a case where a nurse manager was promoted from assistant nurse manager in the same ward, because they had a satisfactory relationship with staff nurses and deeply understood staff nurses’ feelings:

“I got information from the CNS and risk manager. This ward was larger than others in the number of physical restraints…” (ID14).

#### 3.4.3 Judging the problem

Nurse managers confidently determined what problems they should deal with in their wards. Their confidence arose from accurate assessment of considerable information they had collected, their beliefs, and the hospital mission. Nurse managers not only assessed detailed information but also considered the context of complex problems and the essence of problems to grasp the whole situation in the ward. Some nurse managers took time to assess the information until they fully understood the ward situation. One inexperienced nurse manager could not make her own determination because she lacked confidence in her assessment and her own belief:

“Staff nurses worked until 9 p.m…. turnover rate of staff nurses was 50% in this ward… a lot of absenteeism through illness… (I was assigned this ward) firstly, I started to decrease overtime and prevent absenteeism based on my policy” (ID17).

### 3.5 Deciding priorities and solutions

Nurse managers decided the most important problems to be solved based on urgency or impact on patients and staff. Nurse managers thought about how patients and staff nurses would be affected, if the ward situation remained the same. That is to say, nurse managers were proactive about future crises, and then, or simultaneously, they generated ideas and chose solutions.

#### 3.5.1 Prioritizing problems according to impact or urgency

Nurse managers prioritized problems according to impact on patient or staff nurses. For example, a nurse manager dealt with improving high turnover as soon as possible. She thought that if the high nurse turnover rate in the emergency department continued, the ward could no longer admit patients.

In some cases, nurse managers took their time dealing with problems. These cases involved a considerable burden on staff nurses, such as transforming care delivery systems, or having multiple interested parties such as doctors and other professionals. Nurse managers took a long time in preparing to deal with problems:

“I prioritized problems by what I wanted to deal with. Additionally, the time needed to resolve the problem. The problem (constructing a system for discharge planning of CHF patients) involved not only staff nurses but also doctors… I took one year to decide to change it” (ID16).

#### 3.5.2 Choosing appropriate solutions from a variety of options

Nurse managers sought and referred to successful solutions that they gained from experience or heard from superiors or assistant nurse managers. For example, a nurse manager tried to introduce team nursing that had one leader for two teams and members changed between teams every few months because of efficient staffing and active communication among members. A previous nurse manager adopted fixed team nursing with fixed team leaders and members. She thought that team nursing was efficient due to success in introducing this strategy in a previous ward:

“I dealt with many problems as a nurse manager. I was more familiar with problem‐solving approaches than staff nurses because I had experienced more (as a nurse manager) than staff nurses” (ID23).

In addition, nurse managers flexibly adopted these solutions because each ward had different characteristics.

### 3.6 Suggesting solutions in an acceptable way

Nurse managers carefully proposed their ideas for solutions so that staff nurses would not refuse them, and change would be realized. Nurse managers recognized that they should empathize with staff nurses who might be burdened by change or to lead change process successfully. Nurse managers considered staff nurses’ characteristics such as age or years of experience, and the balance of power among staff nurses. This information was gathered from micro and macro perspectives to develop strategies for proposing their ideas.

#### 3.6.1 Involving influential staff explicitly

To communicate their identified problem and chosen solution with staff, nurse managers firstly approached an assistant nurse manager or senior nurses. Nurse managers encouraged them to understand the need to change and deal with change autonomously.

Nurse managers recognized that an assistant nurse manager and senior nurses were influencers in wards because they had power and authority as an assistant nurse manager or a “senpai,” which is like a mentor in wards. Nurse managers brought the influencer on their side to avoid resistance to change from staff nurses. For example, a nurse manager daringly appointed a senior nurse who would be an obstacle to change to a project leader:

“In a previous ward, senior nurses were disturbed. I dared to involve senior nurses, if senior nurses might be disturbed by the change. I dared to ask a nuisance's opinion. Such as how do you think? I think that I firstly got a nuisance on my side” (ID15).

#### 3.6.2 Setting realizable goals

Nurse managers tended to set low to moderate goals for their identified problems, comparing them to their ideal situation of the ward. For example, a nurse manager wanted staff nurses to be eager to study nursing care and new medical devices, but did not impose this ideal on them because some staff nurses lacked motivation to study and others did not have time to study for family reasons. The nurse managers thought that imposition would lead to strong resistance to change and that empathy for staff nurses could achieve change successfully. They judged tolerance levels for change goals according to the whole situation:

“I was in the ICU, an ICU staff nurse seemed eager to study (the latest medical technique or device) … but, (in the Orthopedic surgery unit) staff nurses did not seem eager to do so… I can't constrain them. I could not suggest that staff nurses participate in a study session after work… (in the Orthopedic surgery unit) nurses were married, so I proposed that a study session be conducted after the meeting” (ID10).

Some nurse managers, however, did not abandon their ideal goals but kept them in mind; some nurse managers had strong desires to achieve their ideal goals and prepared themselves to take time to achieve them.

#### 3.6.3 Timing of communicating

Nurse managers considered when to communicate the identified problem and solution to staff nurses. Some nurse managers avoided communicating the problem immediately after being assigned to a new ward because they thought that staff nurses would not accept their suggestion. Some nurse managers waited 2–6 months before making suggestions until they had established good relationships with staff nurses:

“(When I am assigned to a new ward), I don't know much about staff nurses, I feel anxiety about them accepting my thoughts and suggestions… I think the timing of a suggestion is important” (ID8).

However, nurse managers immediately dealt with urgent situations such as high turnover, even if nurse managers had not built good relationships with staff nurses. As a result, improving high turnover led to establishing trust with the nurse manager.

### 3.7 Evaluating change outcomes for the future

Nurse managers assessed change outcomes based on information such as formal data and staff nurses’ attitude to nursing care and the ward atmosphere, gathered through micro and macro perspectives. Some nurse managers conveyed a positive change outcome towards staff nurses and appreciated that the staff nurses’ experience of change was painful. The nurse managers thought that empathy towards staff nurses’ burden might alleviate pain induced by the change.

#### 3.7.1 Feeling the change is successful

Nurse managers evaluated the success of change from data such as turnover numbers, number of physical restraints, and patients’ statements in wards. For example, a nurse manager felt successful about decreasing physical restraint based on ward data and staff nurses’ attitude in daily care:

“The risk manager said to me that the number of physical restraints came down… and I recognized that more staff nurses tried to provide patients with wheelchairs” (ID14).

On the other hand, when goals could not be achieved, nurse managers reflected on the essence of the identified problem or the goal set. Their reflection might contribute to successful changes in the future.

#### 3.7.2 Appreciating the pains of the staff nurses

Some nurse managers gave staff nurses feedback on patients’ or others statements to appreciate the pains staff nurses had taken to bring about change. Because nurse managers understood staff nurses’ hard work during the change and intended to enhance staff nurses’ motivation for future changes. A nurse manager gave positive feedback from patients to staff nurses to increase motivation for change:

“Patients’ statements that staff nurses provided good care were increasing more than ever before… I gave all positive feedback to (staff nurses) … (staff nurses) feel good about it” (ID10).

## 4 DISCUSSION

The findings of this analysis revealed four phases of change processes in wards and that nurse managers’ four attributes contribute to promoting change. When nurse managers identified problems, four attributes, particularly “having both macro and micro perspectives” and “respecting own beliefs and external standards” were indispensable. These results compensate for the lack of previous research that did not include the process used by nurse managers to identify problems. Yoshida et al. (2010) indicated that nurse managers had difficulty conceptualizing about problems in their wards. This research might contribute to resolving their difficulties.

Regarding micro and macro perspectives, management science indicates that these perspectives are important for change efforts to deeply understand situations (Nicholls, 1988). These perspectives may be more important in clinical nursing situations than in business or management because of the very complex situations with multiple interests that occur in a ward. Nurse managers’ micro and macro perspectives might be indispensable for grasping complex situations.

In this study, nurse managers identified the problem to be changed based on their beliefs. The findings were similar to previous research wherein nurse managers’ beliefs created future directions for wards (Bondas, 2009; Salmela, Eriksson, & Fagerstrom, 2012), but the present results extend previous research. These findings emphasize not only the importance of nurse managers’ beliefs but also external standards to identify problems. In this study, some nurse managers kept the hospital mission or rules of the nursing association in mind and attempted to align their own belief with this mission. Nurse managers are responsible for achieving the hospital mission (Yoder‐Wise, 2011). If nurse managers’ beliefs are not consistent with the hospital mission, change cannot be successful. As leaders, nurse managers should have a sense of achieving the hospital mission or following social trends in their attempts to change their ward.

Empathy was an important component of nurse managers’ attributes for promoting change. Previous research indicates that emotional intelligence, which encompasses empathy, promotes change processes (Suhonen & Paasivaara, 2011; Taylor, Roberts, Smyth, & Tulloch, 2015). Nurse managers as middle managers have limited power and authority. In these situations, nurse managers’ empathy might effectively circumvent staff nurses’ resistance to change. In addition, the importance of empathy might be partly influenced by Japanese culture, which emphasizes the need for harmonious relationships (Dorfman, Hibino, Lee, Tate, & Bautista, 1997). Harmonious relationships created by empathy might be important for Japanese change processes.

Based on our results, strategies are suggested to promote leading change in wards. First, leadership programs for nurse managers should be developed to enhance the four attributes, for example, leadership programs cultivating nurse managers’ emotional intelligence. Previous research (Taylor et al., 2015) described effective education programs to improve nurse managers’ emotional intelligence. Second, hospital administrators should create a working environment that enables nurse managers to participate in leadership programs. Nurse managers have difficulty taking long periods off work to participate in such programs (Ibe et al., 2012). The development of organizational support systems for nurse managers is needed to increase leadership. Last, staff nurses should commit to change processes autonomously. In this study, nurse managers struggled with staff nurses’ resistance to change. If many staff nurses have a commitment to the change process, change management will be more successful.

### 4.1 Study limitations

The participants were limited to Japanese nurse managers. Nurse managers’ authority and their education system in Japan are different from other counties. This context may affect their attributes of promoting change, limiting the transferability of our findings to other counties. However, our results may inform the practice of nurse managers in other countries.

## 5 CONCLUSION

This study illustrated nurse managers’ perspectives on the basic processes of leading change in wards. In addition, this research might shed light on the attributes of nurse managers that are necessary for successful change management. Hospital administrators should provide nurse managers with opportunities for leadership training.

## AUTHORS’ CONTRIBUTION

Yoshimi Kodama, Hiroki Fukahori: Study design, Data analysis and Manuscript preparation; Yoshimi Kodama: Data collection.

## CONFLICT OF INTEREST

None
